# Transcatheter Aortic Valve Implantation and Replacement: The Latest Advances and Prospects

**DOI:** 10.3390/jcm14061844

**Published:** 2025-03-09

**Authors:** Milos Brankovic, Abhishek Sharma

**Affiliations:** 1 Cardiovascular Division, Department of Medicine, University of Minnesota, Minneapolis, MN 55455, USA; 2 Division of Cardiology, Department of Medicine, Rutgers New Jersey Medical School, Newark, NJ 07103, USA

**Keywords:** transcatheter aortic valve replacement, TAVR, transcatheter aortic valve implantation, TAVI, trials, update, device technology, valve design, structural heart disease, aortic stenosis

## Abstract

Transcatheter aortic valve replacement (TAVR) has revolutionized the treatment of aortic stenosis, particularly in patients at high risk of adverse events for traditional open-heart surgery. Since the early 2000s, TAVR has evolved rapidly with advancements in device technology, procedural techniques, and patient selection criteria. Over the past 20 years, this catheter-based procedure has significantly improved patient survival and quality of life, demonstrating both the safety and efficacy of TAVR, even in patients at low surgical risk. This paper reviews the latest advances in valve design and strategies for treating aortic stenosis. It explores the challenges with long-term outcomes given the younger age of patients undergoing TAVR and the prospects of emerging technologies to improve long-term outcomes.

## 1. Introduction

Aortic stenosis (AS) is the most common valvular heart disease in the elderly that leads to progressive left ventricular remodeling, heart failure, reduced exercise capacity, and eventually death if left untreated [1]. Traditional surgical aortic valve replacement (SAVR) has long been the standard of care for symptomatic patients with severe AS. However, in many cases, particularly among the elderly or patients with complex medical histories, open surgery is not a feasible option because of heightened perioperative risk due to comorbidities or advanced frailty. In response to this clinical challenge, transcatheter aortic valve replacement (TAVR) was introduced as a less invasive alternative, offering a viable solution to these patients.

Since initial trials showed the superiority of TAVR in patients with severe symptomatic AS and prohibitive and high surgical risk, the field has evolved dramatically. Subsequent trials have demonstrated excellent results with TAVR even among patients with lesser perioperative risk in terms of procedural safety and clinical outcomes [2,3,4]. The most recent trials further expand the evidence by investigating the role of TAVR in asymptomatic patients with severe AS with myocardial fibrosis on cardiac magnetic resonance (CMR) imaging and those with moderate AS and left ventricular dysfunction [5,6,7]. This paper reviews the latest trials and advances in valve design and strategies for native aortic stenosis. Finally, this paper explores the challenges with long-term outcomes given the younger age of patients undergoing TAVR and the prospects of emerging technologies to improve long-term outcomes.

## 2. Discussion

### 2.1. Early Development and Initial Indications

#### TAVR for Symptomatic Severe AS with High and Prohibitive Surgical Risk

Although the concept of TAVR emerged in the 1960s, it was not until the early 2000s that a new horizon began when Dr. Alain Cribier reported the first human case of TAVR in a patient with AS [8]. Early clinical reports have demonstrated feasibility and a procedural success rate of 70–80% in symptomatic patients with severe AS unsuitable for SAVR [9,10]. The PARTNER 1 Cohort B trial was the first randomized controlled trial focusing on inoperable patients, showing a procedural success rate of over 95% with a clear mortality benefit [11]. The study revealed a hazard ratio of 0.51 (95% CI: 0.38–0.68) for 1-year all-cause mortality of the first-generation balloon-expendable SAPIEN valve compared with medical treatment including balloon valvuloplasty. This mortality benefit from TAVR remained at the 5-year follow-up [12]. Following these findings, the PARTNER 1 Cohort A trial showed similar mortality rates between TAVR and SAVR in patients at high surgical risk (1-year follow-up: 24.2% vs. 26.8%, *p* = 0.44; 5-year follow-up: 67.8% vs. 62.4%, *p* = 0.76) [13,14]. In parallel, the CoreValve Extreme-Risk Pivotal trial and the CoreValve High-Risk trial revealed the 1-year mortality benefit of the self-expanding CoreValve in inoperable patients with symptomatic severe AS and those at high surgical risk (TAVR vs. SAVR: 14.2% vs. 19.1%, *p* = 0.04) [15,16]. At three and five years, the mortality rates were similar between the TAVR and SAVR groups (5-year follow-up: TAVR 55.3% vs. SAVR 55.4%, *p* = 0.50) [17,18].

Subsequently, the 2012 European Society of Cardiology (ESC) and the European Association for Cardio-Thoracic Surgery (EACTS) valvular guidelines [19] and 2014 American Heart Association/American College of Cardiology (AHA/ACC) valvular guidelines [20] gave class 1 recommendations for TAVR in patients with symptomatic severe AS and prohibitive risk and class 2A for those with high surgical risk. Supported by multiple TAVR registries [21,22,23], these landmark trials led the 2017 AHA/ACC Focus Update of 2014 valvular guidelines to upgrade the recommendations to class 1 in patients with symptomatic severe AS and high surgical risk [24]. This change caused the rapid adoption of TAVR in clinical practice and marked the beginning of a new era in the management of AS.

### 2.2. Contemporary Trials and Expanding Indications

#### 2.2.1. TAVR for Symptomatic Severe AS Across All Risk Categories

Although TAVR has demonstrated survival benefits in patients with prohibitive or high surgical risk, studies have also highlighted some important limitations of TAVR technology. In particular, studies have identified strokes, vascular complications, complete heart block requiring pacemaker implantation, and paravalvular leak (PVL) as key factors influencing the clinical outcomes in patients undergoing TAVR [25,26,27]. Companies have been refining TAVR technology to address these limitations. With new technologies, trials have shifted their focus from assessing mortality to using a composite endpoint, including stroke, heart failure, and valve-related rehospitalization. This broader approach helped assess the full range of benefits and risks, as TAVR has been increasingly used for patients with lesser surgical risk.

**Figure 1:**
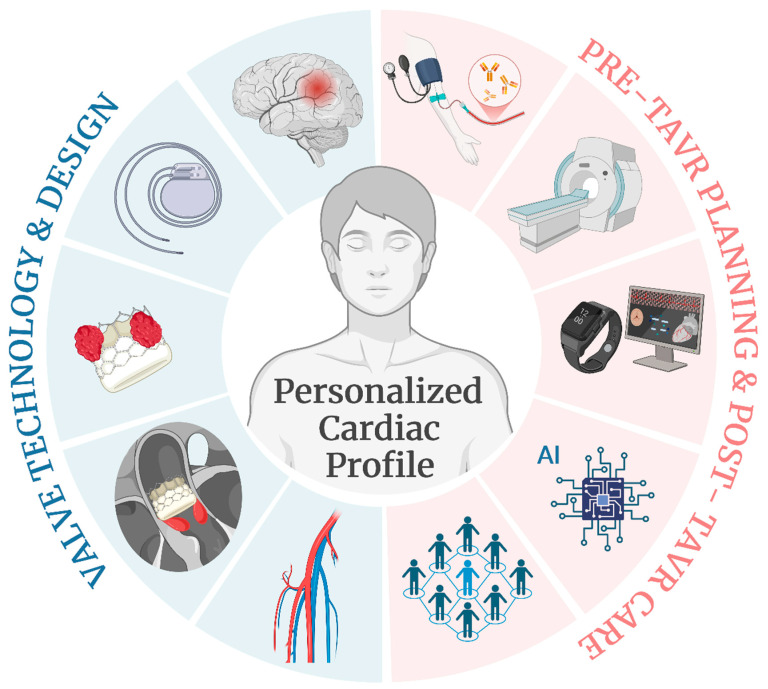
Advances and prospects of TAVR treatment. The future of TAVR will focus on providing care based on the patient’s personalized cardiac profile driven by advances in two key areas: valve technology and design (marked in blue to the left) and pre-TAVR planning with post-TAVR care (marked in red to the right). The first area will be centered on (1) post-procedural stroke prevention, (2) minimizing the need for a permanent pacemaker, (3) valve durability, thrombosis, and calcification, (4) paravalvular leak, and (5) designs to fit a broader range of patient anatomies. The second area will involve a comprehensive care model incorporating (1) patient-specific biomarker data, (2) advances in imaging technologies, (3) telemedicine and wearable technology for remote monitoring, (4) AI and machine learning algorithms to improve patient and device selection, and (5) a multidisciplinary heart team.

**Figure 2:**
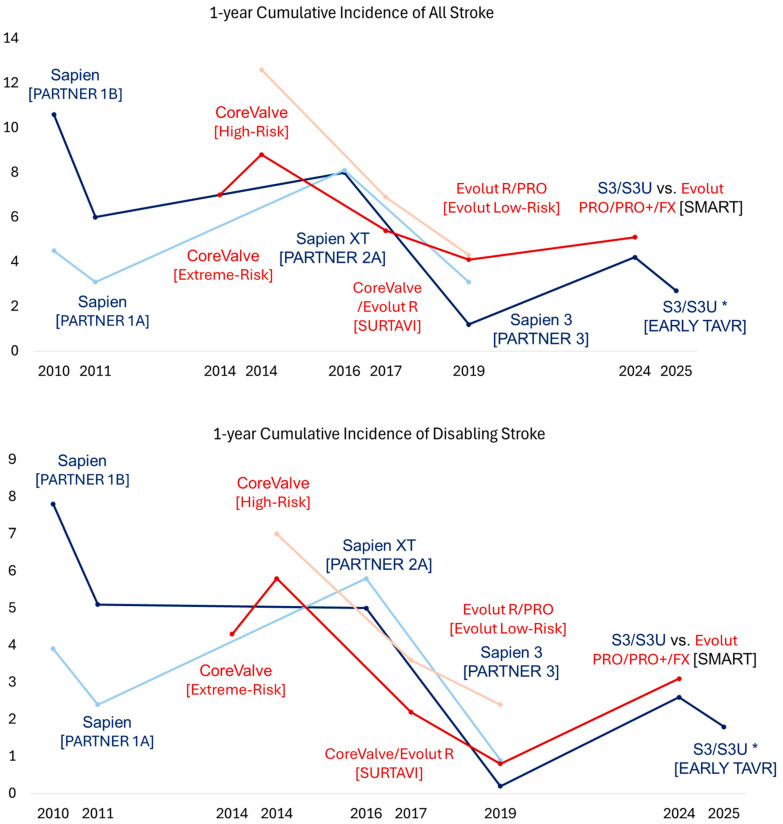
Trend of 1-year cumulative incidences of stroke and disabling stroke across different TAVR trials. The *x* axis denotes the year when the trial was published, and the *y* axis denotes the cumulative incidence as a percentage. The dark blue color denotes the treatment arm receiving SAPIEN TAVR valves, and the light blue color denotes the corresponding treatment arm receiving SAVR. The dark red color denotes the treatment arm receiving Evolut TAVR valves, and the light red color denotes the corresponding treatment arm receiving SAVR. The name of the corresponding trial is presented in parentheses. * Reported 2-year cumulative incidence of stroke and disabling stroke. TAVR, transcatheter aortic valve replacement; SAVR, surgical aortic valve replacement.

**Figure 3:**
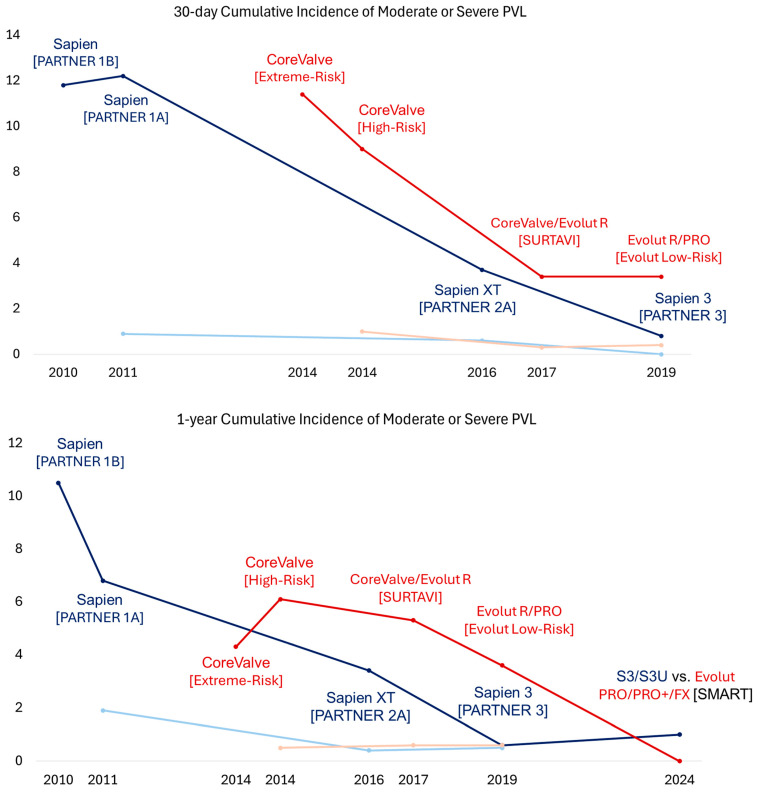
Trend of 30-day and 1-year cumulative incidences of moderate or severe paravalvular leak across different TAVR trials. The *x* axis denotes the year when the trial was published, and the *y* axis denotes the cumulative incidence as a percentage. The dark blue color denotes the treatment arm receiving SAPIEN TAVR valves, and the light blue color denotes the corresponding treatment arm receiving SAVR. The dark red color denotes the treatment arm receiving Evolut TAVR valves, and the light red color denotes the corresponding treatment arm receiving SAVR. The name of the corresponding trial is presented in parentheses. TAVR, transcatheter aortic valve replacement; SAVR, surgical aortic valve replacement; PVL, paravalvular leak.

**Figure 4:**
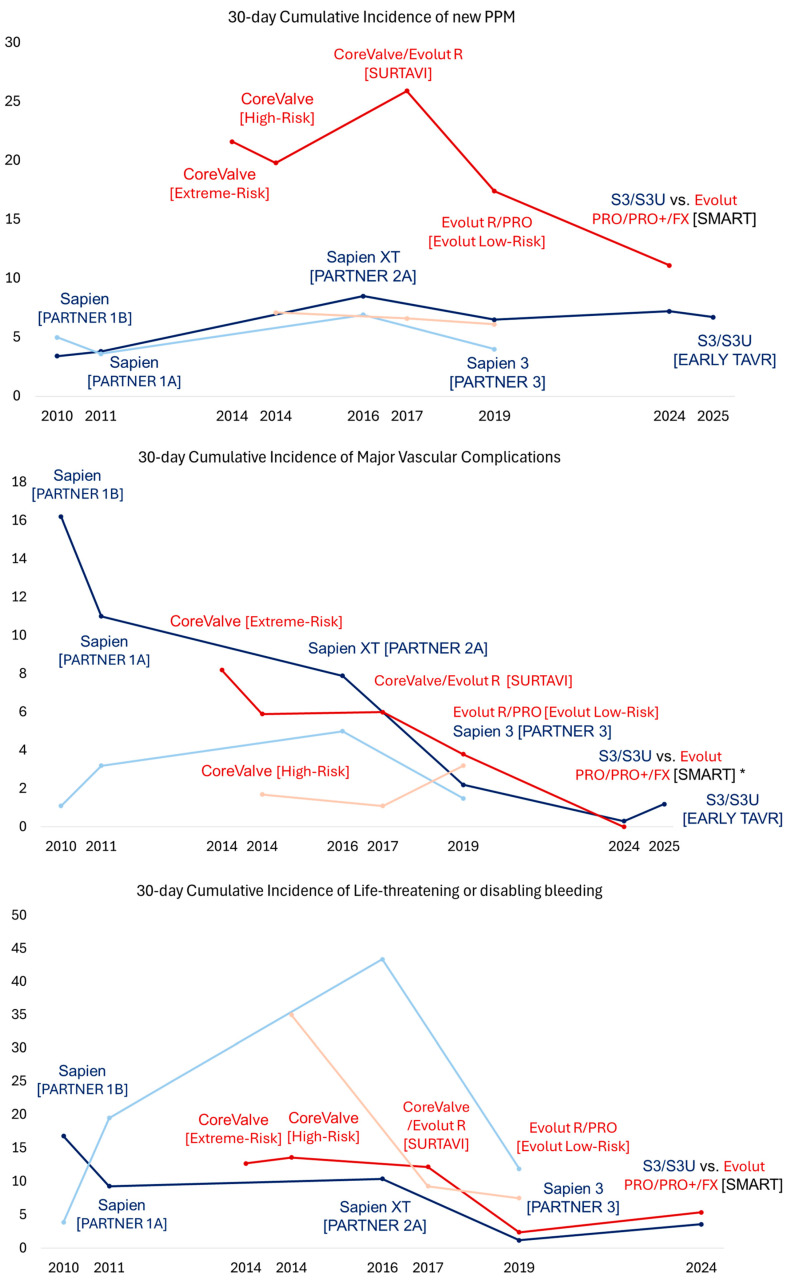
Trend of 30-day cumulative incidence of new permanent pacemaker implantation, major vascular complications, and life-threatening or disabling bleeding across different TAVR trials. The *x* axis denotes the year when the trial was published, and the *y* axis denotes the cumulative incidence as a percentage. The dark blue color denotes the treatment arm receiving SAPIEN TAVR valves, and the light blue color denotes the corresponding treatment arm receiving SAVR. The dark red color denotes the treatment arm receiving Evolut TAVR valves, and the light red color denotes the corresponding treatment arm receiving SAVR. The name of the corresponding trial is presented in parentheses. * Reported 1-year cumulative incidence of major vascular complications. TAVR, transcatheter aortic valve replacement; SAVR, surgical aortic valve replacement; PPM, permanent pacemaker.

In 2016, the PARTNER 2A trial investigated the second-generation balloon-expandable SAPIEN XT valve in patients with symptomatic severe AS and intermediate surgical risk, showing noninferiority to SAVR for a 2-year primary composite endpoint of all-cause mortality or disabling stroke (TAVR vs. SAVR: 19.3% vs. 21.1%, *p* = 0.25) [2]. However, the TAVR group had higher 2-year rates of moderate or severe PVL (8.0% vs. 0.6%, *p* < 0.001) and major vascular complications (8.6% vs. 5.5%, *p* = 0.006) but similar rates of new permanent pacemaker implantation (11.8% and 10.3, *p* = 0.29%) (Figure 1, Figure 2, Figure 3 and Figure 4). The rates of the composite endpoint of death or disabling stroke remained similar at five years (TAVR vs. SAVR: 47.9% vs. 43.4%, *p* = 0.21) [28]. Following these findings, the 2017 AHA/ACC Focus Update gave class 2A recommendations for TAVR as an alternative to SAVR in intermediate-risk patients [24]. In 2017, the SURTAVI trial compared the efficacy and safety of the self-expanding TAVR valve to SAVR in patients with symptomatic severe AS and intermediate surgical risk [4]. In the TAVR group, 84% of subjects received the CoreValve, and 16% received the Evolut R valve. At 2 years, the primary composite endpoint of all-cause mortality or disabling stroke did not differ between the TAVR and SAVR groups (13.2% vs. 14.1%, Bayesian *p* for noninferiority > 0.999). However, the TAVR group had higher 2-year rates of moderate or severe PVL (4.9% vs. 0%, *p* < 0.05), new pacemaker implantation (34% vs. 10%, *p* < 0.05), and aortic valve reintervention (2.5% vs. 0.5%, *p* = 0.002) and 30-day major vascular complications (6% vs. 1%, *p* < 0.05). There was no difference in the rates of clinical valve thrombosis at two years (0.4% vs. 0%, *p* = 0.10). Subsequently, the 2017 ESC/EACTS valvular guidelines gave class 1 recommendations for TAVR in patients with severe symptomatic AS and at least intermediate surgical risk unsuitable for SAVR [29]. At five years, there was no difference in the rates of composite endpoints of death or disabling stroke (TAVR 31.3% vs. SAVR 30.8%, *p* = 0.85) and clinical valve thrombosis (0.7% vs. 0.6%) [30].

The third-generation balloon-expandable SAPIEN 3 (S3) valve was designed to overcome the limitations of the XT valve by incorporating a polyethylene terephthalate (PET) outer skirt as the sealing mechanism to minimize PVL. It also featured a smaller 14–16 Fr delivery system compared to the 18–19 Fr SAPIEN XT system to reduce the risk of vascular complications. In 2019, the PARTNER 3 trial showed the superiority of the S3 valve over SAVR for the 1-year composite outcome of death, stroke, or rehospitalization in patients with symptomatic severe AS and low surgical risk (8.5% vs. 15.1%, *p* = 0.001) [3]. No difference was noted between the TAVR and SAVR groups in the 1-year rates of moderate or severe PVL (0.6% vs. 0.5%, *p* > 0.05), major vascular complications (2.8% vs. 1.5%, *p* > 0.05), new permanent pacemaker implantation (7.3% vs. 5.4%, *p* > 0.05), or clinical valve thrombosis (0.4% vs. 0%, *p* > 0.05). At 5 years, no difference was observed in the rates of the composite endpoint of death, stroke, or rehospitalization (22.8% vs. 27.2%, *p* = 0.07) or bioprosthetic valve failure (3.3% vs. 3.8%) [31]. Notably, the TAVR group had higher rates of valve thrombosis than the SAVR group at five years (2.5% vs. 0.2%, *p* < 0.05). Subsequently, the FDA approved using the S3 valve in patients with symptomatic severe AS across all risk categories. Later, the SAPIEN 3 Ultra (S3U) valve was introduced with a 40% taller PET skirt than the S3 valve. This design modification further reduced the 1-year rates of any PVL (S3U 20% vs. S3 32.4%, *p* < 0.01) with similar moderate to severe PVL rates (2.8% vs. S3: 2.9%) in a propensity score-matched cohort from the S3U registry [32].

In 2019, the Evolut Low-Risk Trial showed the noninferiority of the self-expanding TAVR valve to SAVR for a 2-year primary composite endpoint of all-cause mortality or disabling stroke in patients with symptomatic severe AS and low surgical risk (5.3% vs. 6.7%, Bayesian *p* for noninferiority > 0.999) [33,34]. In the TAVR group, 72% of subjects received the Evolut R valve, and 22% received the Evolut PRO valve. Compared to SAVR, the TAVR group had higher 1-year rates of moderate or severe PLV (3.6% vs. 0.6%) and new pacemaker implantation (19.4% vs. 6.7%) but similar rates of major vascular complications (3.8% vs. 3.5%) and clinical valve thrombosis (0.2% vs. 0.3%). The four-year rates of all-cause mortality, disabling stroke, or aortic valve rehospitalization were lower in the TAVR group (18.0% vs. 14.4%, *p* = 0.04), but the rates of clinical or subclinical valve thrombosis (0.7% vs. 0.6%, *p* = 0.84) and aortic valve reintervention (1.3% vs. 1.7%, *p* = 0.63) were similar. Following these trial results, the 2020 ACC/AHA valvular guidelines gave class I recommendations for transfemoral TAVR over SAVR for symptomatic patients with severe AS regardless of surgical risk if they are older than 80 years old or younger with a life expectancy of less than 10 years. For patients aged 65 to 80 years old, the guidelines gave class I recommendations for either transfemoral TAVR or SAVR after shared decision-making. Of note, TAVR is not recommended for patients younger than 65 [1]. In parallel, the 2021 ESC/EACTS valvular guidelines gave class 1 recommendations for transfemoral TAVR for patients older than 75 years old, those at high surgical risk, or those unsuitable for SAVR. For patients younger than 75 years old with low surgical risk, the guidelines gave class 1 recommendations for SAVR over TAVR. The guidelines gave class I recommendations for transfemoral TAVR or SAVR for the remaining patients according to individual clinical, anatomical, and procedural characteristics [35].

In 2024, a meta-analysis of landmark TAVR trials compared balloon-expandable and self-expanding TAVR valves with SAVR during a long-term follow-up [36]. The study showed no differences in all-cause mortality or disabling strokes between TAVR and SAVR across all risk categories (HR [95%CI]: 1.02 [0.93–1.11]). The study also showed significantly lower valve gradients in patients receiving self-expanding TAVR valves compared with SAVR (mean difference [standard deviation] −2.88 [−3.76 to −2.0]), which was not observed in patients receiving balloon-expandable TAVR valves (*p* for interaction <0.01). However, higher rates of new pacemaker implantation were noted in the patients receiving the self-expanding TAVR valves compared with SAVR (HR [95%CI]: 2.8 [2.3–3.5]). In contrast, higher rates of clinical or subclinical valve thrombosis were noted in patients receiving the balloon-expanding TAVR valves compared with SAVR (4.41% vs. 2.06%, *p* = 0.001), which was not observed in patients receiving self-expanding valves.

#### 2.2.2. TAVR for Symptomatic Severe AS with Bicuspid Aortic Valve or Small Aortic Annulus

It is important to note that patients with bicuspid aortic valves (BAVs) have been excluded from the randomized trials showing the benefit of TAVR in patients with symptomatic severe AS. The current evidence mainly comes from the registry data and non-randomized trials of highly selected patients. The role of TAVR remains uncertain in patients with BAVs given their younger age, often concomitant aortopathy, and implantation challenges. The 2020 ACC/AHA valvular guidelines gave class 2b recommendations for TAVR as an alternative to SAVR in selected patients with BAVs and severe AS [1]. In the recent editorial, Grubb et al. discuss further the current evidence for treating bicuspid AS and provide a framework for future trials [37].

Studies have also suggested that patients with a smaller aortic annulus undergoing TAVR are at a greater risk for impaired valve hemodynamics, prosthesis–patient mismatch, and impaired prosthesis durability [38,39,40]. These findings are of particular interest given the high prevalence of women in this subgroup who have been generally underrepresented in TAVR trials [3,33]. Subsequently, the SMART trial investigated if patients with severe AS and a small annulus have better clinical and hemodynamic outcomes with a self-expanding valve (Evolut PRO/PRO+/FX) or balloon-expandable (SAPIEN S3/S3U) valve. No difference was seen in the primary composite endpoint of death, stroke, or heart failure hospitalization at one year (total cohort: 9.4% vs. 10.6%, *p* < 0.001 for noninferiority; women 9.4% vs. 11.8%, *p* = 0.38) [41,42]. There was also no difference in clinical valve thrombosis at one year (0.3% vs. 0.3%). At one year, self-expanding valves had a lower incidence of bioprosthetic valve dysfunction, defined as the composite endpoint of mean gradient ≥ 20 mmHg, severe prosthesis–patient mismatch, or at least moderate aortic regurgitation, thrombosis, endocarditis, or reintervention compared with balloon-expandable valves (total cohort: 9.4% vs. 41.6%, *p* < 0.001; women: 8.4%. vs. 41.8%, *p* < 0.001).

#### 2.2.3. TAVR for Asymptomatic Severe or Moderate AS

The ongoing research focuses on the feasibility of early AS treatment to prevent or slow cardiac remodeling and functional deterioration and to prevent death in patients with asymptomatic severe or moderate AS. Two recent surgical randomized trials, RECOVERY [43] and AVATAR [44], showed a reduction in all-cause mortality with early SAVR compared with watchful waiting in asymptomatic patients with very severe or severe AS. Given the limited sample size of these trials, a large-scale pragmatic randomized trial, the EASY AS trial (NCT04204915), is underway to confirm if early SAVR reduces clinical events and is cost-effective compared with watchful waiting in this population [45].

To extend these findings to the TAVR population, the recent EARLY TAVR trial investigated if early TAVR with balloon-expandable S3 or S3U would be beneficial over clinical surveillance in asymptomatic patients with severe AS and preserved ejection fraction for the composite endpoint of death, stroke, or unplanned rehospitalization [6]. Notably, 87% of patients in the clinical surveillance group received TAVR during the median of 3.8 years, with almost half of them receiving TAVR within the first year. It remains unclear if the exact reason for such a rapid cross-over was related to the progression of AS from the asymptomatic to the symptomatic phase or it was also biased by the patients’ awareness of potentially lethal cardiac disease. Nonetheless, the intention-to-treat analysis showed a marked benefit of the early TAVR strategy compared with mainly late intervention in clinical surveillance for the composite outcome (26.8% vs. 45.3%, *p* < 0.001), primarily driven by rehospitalization. Although the rates of death and stroke were numerically lower in the early TAVR group, they did not reach statistical significance.

Furthermore, a recent EVOLVED trial investigated if early SAVR or TAVR is superior to conservative management in asymptomatic patients with severe AS, preserved LVEF, and CMR-confirmed mid-wall myocardial fibrosis [7]. The initial screening involved patients with severe AS and either elevated troponin or left ventricular hypertrophy on electrocardiograms who subsequently underwent CMR imaging. Unlike the EARLY TAVR trial protocol, which used exercise testing to confirm no symptoms, the patients enrolled in the EVOLVED trial were classified as asymptomatic based on the physical exam. During a median of 3.5 years, no difference was found between the early and late intervention groups for the primary composite endpoint of death or unplanned aortic stenosis-related rehospitalization (18% vs. 23%, *p* = 0.44). There was an improvement in the prespecified secondary endpoints, such as unplanned aortic stenosis-related hospitalization and the New York Heart Association functional class, in patients who underwent early intervention.

The TAVR UNLOAD trial investigated the benefit of early TAVR in patients with moderate AS and heart failure with a reduced ejection fraction on guideline-directed medical therapy [5]. During a median of two years, no difference was found between early TAVR and clinical surveillance for the primary endpoint of the hierarchical occurrence of death, disabling stroke, disease-related hospitalizations, and heart failure hospitalization equivalents (a win ratio of 1.31 [95% CI:] 0.91–1.88). Furthermore, an ongoing PROGRESS trial (NCT04889872) is enrolling patients older than 65 years, with moderate AS and at least one additional risk factor, to evaluate the safety and effectiveness of the SAPIEN S3, S3U, and S3U RESILIA valves compared with clinical surveillance in patients with moderate AS. Similarly, the EXPAND TAVR II trial (NCT05149755) is currently enrolling patients with moderate AS to test the safety and effectiveness of early TAVR treatment using the Evolut FX and Evolut PRO+ valves.

### 2.3. Advances in Valve Design

Since the early TAVR days, significant advancements in device technology, procedural techniques, and imaging have enhanced both the short- and long-term results of TAVR in AS. The focus of newer-generation TAVR valves includes improving hemodynamic performance, reducing complications such as valve malposition and PVL, enhancing adaptability to various annular shapes, diameters, and calcification patterns, and making valve systems with smaller delivery sheaths accessible for smaller or tortuous calcified vessels. Additionally, modern valves feature more durable materials, such as specially treated tissue or polymeric materials designed to improve leaflet durability and resistance to calcification. New valve designs are also exploring polymer coatings of the valve to reduce the risk of valve thrombosis and improve valve hemodynamics [46].

Of note, studies have also investigated the role of different cerebral embolic protection devices (CEPDs) in reducing the risk of peri-procedural stroke in patients undergoing TAVR [47,48,49,50,51,52]. Nonetheless, recent collective evidence suggests no clear benefit of reducing clinical, neurological, and safety outcomes in this patient population [53].

Commercially available TAVR valves have been classified into self-expanding and balloon-expandable valves. Traditionally, self-expanding supra-annular valves provide a larger effective orifice area and lower gradient. They also tend to be associated with a greater need for new pacemaker implantation than balloon-expandable valves. In contrast, balloon-expandable valves are related to a higher risk of late clinical or subclinical valve thrombosis but allow for more precise positioning and deployment. The final decision between the two systems will depend on the patient’s age, life expectancy, the severity of AS, the anatomy of the aortic annulus, and other risk factors. We provide here an overview of the most recently FDA-approved TAVR valves, which is accompanied by Supplemental Table S1 summarizing TAVR trials supporting the use of these devices.

#### 2.3.1. Edwards SAPIEN Series and RESILIA Technology

The Edwards Lifesciences SAPIEN 3 Ultra valve (FDA-approved in 2024) is a balloon-expandable valve that builds on the success of the original SAPIEN 3 platform. Compared to SAPIEN 3, SAPIEN 3 Ultra has a higher outer textured PET skirt for a better sealing mechanism to reduce PVL. The SAPIEN 3 Ultra valve is available in 20, 23, and 26 mm valve sizes, whereas the standard SAPIEN 3 valve also includes a 29 mm valve size. SAPIEN 3 Ultra and SAPIEN 3 use the same 14 Fr and 16 Fr low-profile delivery systems for smaller arteries. The Edwards SAPIEN 3 (FDA-approved in 2015) is one of the most used TAVR valves. Using a balloon-expandable intra-annular design, the SAPIEN 3 valve features a cobalt–chromium frame with bovine pericardial tissue leaflets. It is used for high-risk, intermediate-risk, and, more recently, low-risk patients.

An ongoing ALLIANCE trial (NCT05172960) is investigating the efficacy and safety of the newest SAPIEN X4 valve (not yet FDA-approved) in patients with severe AS across all risk categories. This new design of the SAPIEN X4 valve features an adjustable frame size for different aortic anatomy, a shorter frame height, a large cell size for easier coronary access, a 14 Fr delivery system (for 23, 26, and 29 mm valve size), and an enhanced outer PET skirt to reduce PVL compared to its predecessors.

The RESILIA valve features bovine pericardial tissue leaflets specially treated with proprietary RESILIA tissue technology to improve durability by preventing early valve deterioration and calcification. This feature may be especially important for younger patients who may otherwise need replacement after five to ten years with traditional bioprosthetic valves. Since RESILIA is a newer material, there are less long-term data to confirm these properties. However, the COMMENCE trial showed excellent 7-year results for RESILIA technology used for the surgical INSPIRIS bioprosthetic aortic valve [54]. RESILIA tissue has been used in the SAPIEN 3 Ultra and SAPIEN X4 valve platforms.

#### 2.3.2. Medtronic CoreValve Evolut Series

The Evolut platform features a repositionable and recapturing delivery system to provide greater precision in valve positioning. The Evolut valve (Medtronic, Minneapolis, MN, USA) uses porcine pericardial tissue leaflets, which have been used in surgical valves for several decades with a well-established track record. The Evolut valves also feature a self-expanding supra-annular nitinol frame with an adaptive porcine pericardial sealing skirt designed to create a better seal at the annular plane to reduce PVL. Evolut valves are also designed to allow post-dilatation in cases where a balloon can expand the valve after deployment to achieve optimal fit.

The Evolut FX+ valve (FDA-approved in 2024) is the latest version of the Evolut TAVR valve with an updated diamond-shaped frame with a cell size larger than that of the Evolut FX and PRO+ valves to offer easier coronary access. Evolut FX and FX+ feature a tall porcine pericardial sealing skirt with an external wrap to reduce PVL and an improved nose cone design for smoother femoral artery insertion via a 14 Fr (for 23, 26, and 29 mm valve) and 18 Fr delivery system (for 34 mm valve) and an InLine sheath. The valves also feature three radiopaque markers to denote commissural locations for improved commissural alignment and a more symmetric deployment. These valves have been approved in symptomatic patients with severe AS across all risk categories.

The Evolut PRO+ valve (FDA-approved in 2017) is an updated version of the Evolut R valve, incorporating a refined self-expanding nitinol frame with porcine pericardial tissue leaflets, a sealing skirt with additional external porcine pericardial wrap, and increased flexibility in deployment. It also features a low 14 Fr delivery profile (23, 26, and 29 mm valves) and an 18 Fr delivery system (34 mm valves) for patients with smaller access vessels. Compared to the Evolute R (FDA-approved in 2015), the Evolut PRO valve has an additional external pericardial wrap for 23–29 mm valves (not 34 mm) to reduce PVL rates, a repositionable mechanism for deployment adjustment, and a lower-profile delivery system to reduce the risk of vascular complications. The EXPAND TAVR II trial is currently enrolling patients with moderate AS to test the safety and effectiveness of early TAVR treatment using the Evolut FX and Evolut PRO+ valves.

#### 2.3.3. Abbott Portico Navitor Systems

The Portico Navitor valve (FDA-approved in 2023) is a self-expanding TAVR valve using a radiopaque nitinol frame (Abbott Vascular, Santa Clara, CA, USA) with an intra-annular design and large cell design to facilitate coronary access. Compared to its predecessor, the Portico valve, Navitor has NaviSeal cuffs synchronized to the cardiac cycle to reduce PVL. The NaviSeal cuffs help close calcification-related gaps between the valve and native annulus by filling these gaps during diastole with blood. The Portico Navitor valve also features an improved low-profile 14 Fr and 15 Fr FlexNav delivery system for patients with limited peripheral access. The Portico Navitor valve is available in 23, 25, 27, and 29 mm sizes. It also allows for repositioning and retrieval during the procedure to ensure optimal placement. Following the favorable results of the PORTICO NG study, the Navitor valve has been FDA-approved in patients with aortic stenosis who are at high or greater risk for surgical mortality [55].

The VANTAGE Trial (NCT04788888) is currently enrolling patients to assess the safety and effectiveness of the Navitor valve in symptomatic patients with severe AS who are at intermediate and low surgical risk. The Abbott Portico valve (FDA-approved in 2021) is a self-expanding predecessor of Navitor, designed with a bovine pericardial valve within a nitinol frame.

#### 2.3.4. Boston Scientific ACURATE Neo2 and LOTUS Series

The ACURATE Neo2 (not FDA-approved) is the latest Boston Scientific (Boston Scientific, Marlborough, MA, USA) self-expanding valve designed with a low-profile delivery system for easier vascular access and a supra-annular position with an open upper frame for easier coronary access. Compared to the prior ACURATE Neo system, the ACURATE Neo 2 features a unique triple-seal design with porcine pericardial tissue to reduce PVL. It also uses three stabilizing arches for axial alignment and upper and lower anchoring crowns for accurate positioning during deployment. Notably, the recent ACURATE IDE trial (NCT03735667) failed to show the noninferiority of the ACURATE Neo2 valve compared to the SAPIEN and Evolut valves for a 1-year endpoint composed of death, stroke, and rehospitalization (14.8% vs. 9.1%). The Lotus Edge was another TAVR valve that was approved by the FDA in 2019 for patients with severe aortic stenosis who were at high risk for surgery. However, the manufacturer discontinued the Lotus Edge in 2020 due to issues with the delivery system.

#### 2.3.5. JenaValve

The Jena Trilogy TAVR valve (JenaValve, Munich, Germany) is a newer TAVR system that features a self-expanding nitinol frame with a supra-annular design, porcine pericardium tissue leaflets, a large cell design for easier coronary access, locator technology for alignment with native anatomy, and a sealing ring for anchoring and annular conformability. The Jena Trilogy valve allows repositioning and retrieval during the procedure to ensure optimal placement. The Jena Trilogy TAVR valve is available in 23, 25, and 27 mm sizes using an 18 Fr delivery system. Compared to other TAVR systems, JenaValve is specifically designed for patients with native aortic regurgitation. The most recent ALIGN-AR trial has demonstrated the promising safety and effectiveness of the Jena Trilogy in symptomatic patients with moderate or greater aortic regurgitation who are at high risk for surgical mortality [56]. JenaValve also received CE mark approval in Europe for testing in patients with severe symptomatic aortic stenosis or aortic regurgitation and high surgical risk.

### 2.4. Personalized Profiles and Multidisciplinary Care

The future of TAVR will increasingly involve personalized approaches tailored to the individual patient’s anatomy and cardiac profile. Advances in genomics, proteomics, cardiac imaging, and artificial intelligence are expected to provide further insights into patient-specific cardiac profiles and help refine patient selection, procedural planning, and post-procedural care. Studies like the EVOLVED trial have moved from traditional AS staging using predominantly echo parameters and started incorporating remodeling data to better stratify patients who would benefit from TAVR. As TAVR technology evolves, integrating a personalized approach and comprehensive care models will be key to optimizing TAVR-related outcomes. Therefore, a multidisciplinary heart team approach will continue to be essential to assess each patient’s suitability for TAVR, determine the most appropriate intervention, and manage post-procedural care.

Identifying genetic factors and biomarkers associated with a patient’s response to TAVR or determining the urgency of TAVR has been actively explored [57]. Such a biomarker-guided approach might also help explore the role of sex-related physiological differences in the molecular pathways and different outcomes in women and men [58]. Studies to understand how an individual’s biomarkers affect the likelihood of complications are underway and would allow for more precise interventions and outcomes. Advanced imaging technologies (such as CT and MRI) already allow for precise assessments of the aortic valve, coronary anatomy, and vascular access. Integrating AI and machine learning algorithms could further enhance these assessments, enabling the selection of the most suitable TAVR device and procedure approach based on each patient’s unique anatomy [59]. Several fully automated software tools with demonstrated high correlation coefficients for most TAVR-related measurements are already available [60]. Yet, further research is needed before implementing such algorithms in clinical practice.

Exploring a patient’s frailty and comorbid conditions may significantly influence their recovery and overall prognosis by addressing their unique needs. For example, studies have investigated the use of wearable devices and smartphones to assess objective patient-reported outcomes [61]. Notably, a study showed an excellent compliance of 80% in elderly, frail TAVR patients. Furthermore, Liu and colleagues showed that using wearable smartwatches (HUAWEI Technologies Co., Ltd., Shenzhen, China) at home in patients who underwent TAVR enabled the remote detection of conduction abnormalities, leading to a change in management within 30 days post procedure [62]. The use of telemedicine and wearable technology [63] for the continuous remote monitoring of TAVR patients provides a great opportunity for the early detection of complications, improved patient engagement, and better long-term management. Studies are underway to provide evidence for the most effective way to implement such technologies in practice.

Following the success of TAVR in patients with native AS, the use of TAVR as a valve-in-valve (ViV) procedure has expanded to treating failed surgical or percutaneous valves. Non-randomized data have suggested promising 30-day and 1-year outcomes of ViV TAVR compared to traditional open redo SAVR in high-risk surgical candidates [64,65]. However, ViV TAVR is limited to patients with large enough annuli and carries a heightened risk of coronary obstruction. To address the latter issue, the Bioprosthetic or native Aortic Scallop Intentional Laceration to prevent Iatrogenic Coronary Artery obstruction during TAVR (BASILICA) trial investigated the use of the BASILICA technique to split the leaflet and reduce the risk of iatrogenic coronary obstruction [66]. The investigators showed the feasibility of this technique both in native and bioprosthetic valves. Studies have also suggested that BASILICA may help reduce the risk of valve thrombosis by enhancing sinus washout [67]. However, good-quality randomized clinical trials are still pending to address the use of ViV TAVR in this patient population. Given that ViV TAVR presents an attractive alternative to redo SAVR, future developments in TAVR design should focus on optimizing the benefits of this procedure for patients with bioprosthetic valve failure.

Finally, the costs of TAVR devices and related hospitalizations play a critical role in enhancing the procedure’s applicability and sustainability. Understanding the financial burden of both can help ensure equitable access to this life-saving treatment. By evaluating device costs and hospital stays, which can vary in terms of technology and materials, healthcare teams can identify cost-effective, high-quality options. Sustainable practices like shorter hospital stays, less peri-procedural complications, and optimized resource use can improve TAVR scalability, making it more widely available to patients in need worldwide.

## 3. Conclusions

With advances in device technology, procedural techniques, and imaging, TAVR has become safer, more effective, and applicable to a wider range of patients. As a result, different generations of valves may not be directly comparable due to differences in technology and outcomes. Therefore, caution is advised when combining results from newer TAVR valves with those from older ones, as the results from one generation may not be applicable to the other.

Ongoing challenges, such as device durability and long-term complication management, have been addressed to improve the long-term quality of life for those affected by AS. The future of TAVR valve designs is centered on improving these outcomes by addressing challenges like valve durability and thrombosis, PVL, complete heart block requiring a pacemaker, and the ability to treat a broader range of patient anatomies.

In parallel, the clinical paradigm of managing AS is changing. Traditionally, the severity of AS was primarily assessed using echocardiography, but now, additional tools such as computed tomography (CT) or CMR imaging and biomarkers are being used to understand cardiac remodeling and better guide treatment decisions. As the field continues to evolve, expanding the indications for TAVR to lower-risk populations, intervening before irreversible cardiac damage occurs, and incorporating personalized approaches will be key focus areas.

## Data Availability

Publicly available data were used for this study. Please see the reference list.

## Supplementary Materials

The following supporting information can be downloaded at https://www.mdpi.com/article/10.3390/jcm14061844/s1, Table S1: Summary of past and incoming clinical trials of transcatheter aortic valve replacement.

## Abbreviations

The following abbreviations are used in this manuscript:

AS	Aortic stenosis
PVL	Paravalvular leak
SAVR	Surgical aortic valve replacement
TAVR	Transcatheter aortic valve replacement
CMR	Cardiac magnetic resonance
PET	Polyethylene terephthalate
FDA	Food and Drug Administration
