# Supplementary material for: Transcatheter Aortic Valve Implantation and Replacement: The Latest Advances and Prospects
Source: J Clin Med. 2025 Mar 9;14(6):1844. doi: 10.3390/jcm14061844 (PMC11942769; doi:10.3390/jcm14061844)
Supplement: Supplementary file 1 [file jcm-14-01844-s001.zip › jcm-3435654-supplementary.pdf]

Supplemental Table. Summary of past and incoming clinical trials of transcatheter aortic valve replacement.

Trial (n)	Year	STS score Mean ± SD	Valve	Type	AS	Primary Endpoint	Major Results
**PARTNER 1B** (TAVR: 179, ST: 179)	2010	Inoperable[a]	Sapien	b-e	S/S	All-cause death	1-year PE: TAVR 30.7% vs. ST 50.7%, p<0.001
**PARTNER 1A** (TAVR: 348, SAVR: 351)	2011	11.8 ± 3.3	Sapien	b-e	S/S	All-cause death	1-year PE: TAVR 24.2% vs. SAVR 26.8%, p=0.44<br>5-year PE: TAVR 67.8% vs. SAVR 62.4%, p=0.76)
**CoreValve Extreme-Risk** (TAVR: 489)	2014	Inoperable[a]	CoreValve	s-e	S/S	All-cause death or major stroke	1-year PE: 26%<br>1-year all-cause death 24%
**CoreValve High-Risk** (TAVR: 394, SAVR: 401)	2014	7.3 ± 3.3	CoreValve	s-e	S/S	All-cause death	1-year PE: TAVR 14.2% vs. SAVR 19.1%, p=0.04<br>5-year PE: TAVR 55.3% vs. 55.4%, p=0.50
**PARTNER 2A** (TAVR: 1011, SAVR: 1021)	2016	5.8 ± 2.1	Sapient XT	b-e	S/S	All-cause death or disabling stroke	2-year PE: TAVR 19.3% vs. 21.1%, p=0.25<br>5-year PE: TAVR 47.9% vs. SAVR 43.4%, p=0.21
**SURTAVI** (TAVR: 864, SAVR: 796)	2017	4.4 ± 1.5	CoreValve / Evolut R[b]	b-e	S/S	All-cause death or disabling stroke	2-year PE: TAVR 13.2% vs. SAVR 14.1%, Bayesian p for noninferiority >0.999<br>5-year PE: TAVR 31.3% vs. SAVR 30.8%, p=0.85
**PARTNER 3** (TAVR: 496, SAVR: 454)	2019	1.9 ± 0.7	Sapien 3	b-e	S/S	All-cause death or disabling stroke or rehospitalization	1-year PE: TAVR 8.5% vs. 15.1%, p-0.001<br>5-year PE: TAVR 22.8% vs. 27.2%, p=0.07
**Evolut Low-Risk** (TAVR: 725, SAVR: 678)	2019	1.9 ± 0.7	Evolut R / PRO[c]	s-e	S/S	All-cause death or disabling stroke	2-year PE: TAVR 5.3% vs. 6.7%, Bayesian p for noninferiority >0.999<br>4-year PE: 10.7% vs. 14.1%

**SMART[d] (s-e: 355, b-e: 361)**	2024	3.3 ± 1.9	Evolut PRO/PRO+/FX Sapien S3 / S3 Ultra	s-e / b-e	S/S	PE 1: All-cause death or disabling stroke or rehospitalization for heart failure PE 2: bioprosthetic-valve dysfunction[e]	1-year PE 1: s-e 9.4% vs. b-e 10.6, p<0.001 for non-inferiority 1-year PE 2: s-e 9.4% vs. 41.6%, p<0.001
**EARLY TAVR (TAVR 455, CS 446)**	2025	1.8 ± 1.0	Sapient 3 / S3 Ultra	b-e	A/S	All-cause death or stroke or unplanned hospitalization for cardiovascular causes	1-year PE: TAVR 26.8% vs. 45.3%, p<0.001
**EVOLVED** (TAVR/SAVR 113, CM 111)[f]	2025				A/S/CMR+[g]	All-cause death or unplanned aortic stenosis–related hospitalization	1-year PE: TAVR/SAVR 18% vs. CM 23%, p=0.44)
**TAVR UNLOAD** (TAVR 89, CASS 89)	2025	4.0 ± 2.6	Sapien 3	b-e	A/M[h]	Hierarchical occurrence of: 1) all-cause death; 2) disabling stroke; 3) disease-related hospitalizations and HF hospitalization equivalents; and 4) the change from baseline in the KCCQ-OSS.	23-months PE: TAVR wins in 47.6% of pairs vs. CASS 36.6% of pairs, p=0.14
**PROGRESS**	Estimated 2029		Sapien 3 / S3 Ultra / S3 Ultra RESILIA	b-e	A/M		Recruiting
**EXPAND TAVR II**	Estimated 2026		Evolut FX / PRO+	s-e	A/M		Recruiting
**ALLIANCE**	Estimated 2025		Sapien X4	b-e	S/S		Active, not recruiting
**PORTICO NG (TAVR 120)**	2023	4.0 ± 2.0	Navitor	s-e	S/S	All-cause death or moderate or greater PVL	30-day PE: 0% 1-year all-cause death: 4.6% 1-year disabling stroke: 0.8% 1-year PVL: 1%
**VANTAGE**	Estimated 2025		Navitor	s-e	S/S		Recruiting
**ACURATE IDE**	2024		Acurate neo2	s-e	S/S	All-cause death or stroke or rehospitalization	1-year PE: Acurate neo2 14.8% vs. Sapien/Evolute 9.1%
**ALIGN-AR** (TAVR 180)	2024		Jena Trilogy	s-e	S/AR	All-cause death	1-year PE: 7.8%, p non-inferiority <0.001

[a] Inoperable was defined as 30-day mortality or serious irreversible condition of at least 50% post-surgery.
[b] In the TAVR arm, 84% of subjects received the CoreValve, and 16% of subjects received the Evolut R valve.

[c] In the TAVR arm, 72% of subjects received the Evolut R valve, and 22% of subjects received the Evolut PRO valve.
[d] In the SMART trial, subjected with symptomatic severe aortic stenosis and an aortic-valve annulus area of 430 mm^2 or less who underwent TAVR were compared between ballon-expandable and self-expanding valves.
[e] In the SMART trial, investigators reported two co-primary endpoints including (1) clinical outcome composed of death, disabling stroke, or rehospitalization for heart failure, and (2) bioprosthetic-valve dysfunction outcome composed of hemodynamic structural valve dysfunction, defined as an aortic-valve mean gradient of 20 mm Hg or higher; nonstructural valve dysfunction, defined as severe prosthesis–patient mismatch or at least moderate total aortic regurgitation; clinical valve thrombosis; endocarditis; or aortic-valve reintervention
[f] In the EVOLVED trial, 75% of subjected underwent SAVR and 25% underwent TAVR in the early intervention arm.
[g] In the EVOLVED trial, investigators enrolled patients with asymptomatic severe aortic stenosis, left ventricular ejection fraction >50%, and cardiac magnetic resonance confirmed myocardial fibrosis.
[h] In the TAVR UNLOAD trial, investigators enrolled patients with moderate aortic stenosis and left ventricular ejection fraction 20-50% on guideline-directed medical therapy.
AR, aortic regurgitation. AS, aortic stenosis. A/S, asymptomatic/severe. B-e, ballon-expandable. CASS, clinical aortic stenosis surveillance. CM, conservative management. CS, clinical surveillance. S-e, self-expanding. S/S, symptomatic severe aortic stenosis. PE, primary endpoint. PVL, paravalvular leak. ST, standard therapy.
